# Supplementary material for: Additive manufacturing of multi-material and hollow structures by Embedded Extrusion-Volumetric Printing
Source: Nat Commun. 2025 Jul 22;16:6730. doi: 10.1038/s41467-025-62057-6 (PMC12284217; doi:10.1038/s41467-025-62057-6)
Supplement: Supplementary file 1 — Supplementary information [file 41467_2025_62057_MOESM1_ESM.pdf]

Supplementary Information

Additive manufacturing of multi-material and hollow structures by Embeddded Extrusion-Volumetric Printing

Silvio Tisato[1], Grace Vera[1], Qingchuan Song[2,3], Niloofar Nekoonam[1,2], Dorothea Helmer[1,2,3]*

[1] Freiburg Materials Research Center (FMF), University of Freiburg, Stefan-Meier-Str. 21, 79104 Freiburg im Breisgau, Germany

[2] IMTEK - Laboratory of Process Technology, University of Freiburg, Georges-Köhler-Allee 103, 79110 Freiburg im Breisgau, Germany

[3] Freiburg Center of Interactive Materials and Bioinspired Technologies (FIT), University of Freiburg, Georges-Köhler-Allee 105, 79110 Freiburg, Germany

Structure	EMB3D Time [s]	TVAM Time [s]	Total print time [s]
2-Arm helix (1d)	~180	109	289
3Bellow (1i)	180	142	322
2Bellow (1j)	~170	144	314
Thinker (2a)	~120	151	271
Skeleton sphere (2b)	~180	162	342
Hollow Cylinder (2e)	~9	122	131
Lattice (2g)	~180	108	288
Bending Bellow (2k)	~90	155	245
Cylindrical chip (3c)	~12	121	133
Flat chip (3g)	~8	148	156

Supplementary Table 1. Print times of the embedded 3D printing and volumetric printing steps as well as combined print time for the parts shown in Figure 1, Figure 2 and Figure 3. For additive ETVAM, the addition of a second material increases print times significantly, but all parts still complete in less than 6 minutes, while for subtractive ETVAM, the embedding time of the channel is significantly faster than the TVAM step, leading to only a slight increase in print times.

	EmVP	Vat exchange overprinting	Positive EmVP (this work)
Place different materials in the bulk of the part	Yes	Yes	Yes
Place different materials on the surface of the part	No	Yes	Yes
Matrix needs to be engineered to possess supporting properties	Yes	No	Yes
Requires at least one ink to be extrudable	Yes	No	Yes
Requires matched curing properties of the individual materials	No	No	Yes
Need to align structures in supporting material	No	Yes	No
Simultaneous shaping of multiple materials by TVAM	No	No	Yes
Single projection set	Yes	No (one set per material)	Yes
Requires EMB3D printer	Yes	No	Yes
Requires support structures to aid alignment	No	Yes	No
Requires multi-wavelength projector to perform alignment	No	Yes	No
Need to perform material exchange	No	Yes	No
High positioning freedom for secondary material	Yes (no surface)	No (only unoccupied areas)	Yes (surface and bulk)

Supplementary Table 2. Comparison of multi-material methods developed for TVAM, showing advantages and disadvantages.

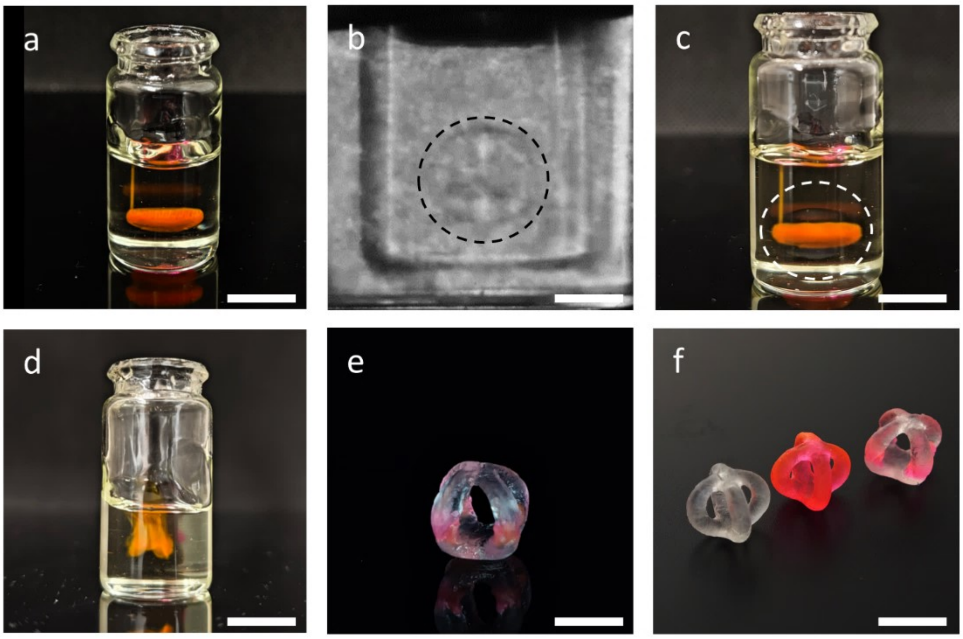

Supplementary Figure 1. Print process for positive EmVP and Comparison of prints in Mat 1, Mat 2 and combination of the two shown on prints of a spherical cage structure and showing proof that this approach is distinguished from EmVP overprinting. (a) Deposited Mat 1 in Mat 2 before curing. Note how the area of the deposited disk is fully filled. (b) Shadowgram of the positive EmVP of the spherical cage structure during the TVAM step. (c) Cured structure spanning across material boundaries. Note how the diameter of the printed structure is lower than that of the deposited disk of Mat 1. Thus, distinguishing this method from previously shown overprinting approaches. (d) After the structure is removed there is leftover Mat 1 in the vial, further proving that only the desired areas of Mat 1 are cured and that Mat 1 is not fully immersed in Mat 2 after the print and hence this approach is not characterized as overprinting. (e) Printed multi-material structure extracted from the vial. (f) Visual comparison of structures printed by TVAM in a single material vial of Mat 2 (left), Mat 1 (center) and a combination of the two (right). Scale bars: a,d 12 mm, b 5.5 mm, c 10.5 mm, e 6 mm, f 7.5 mm

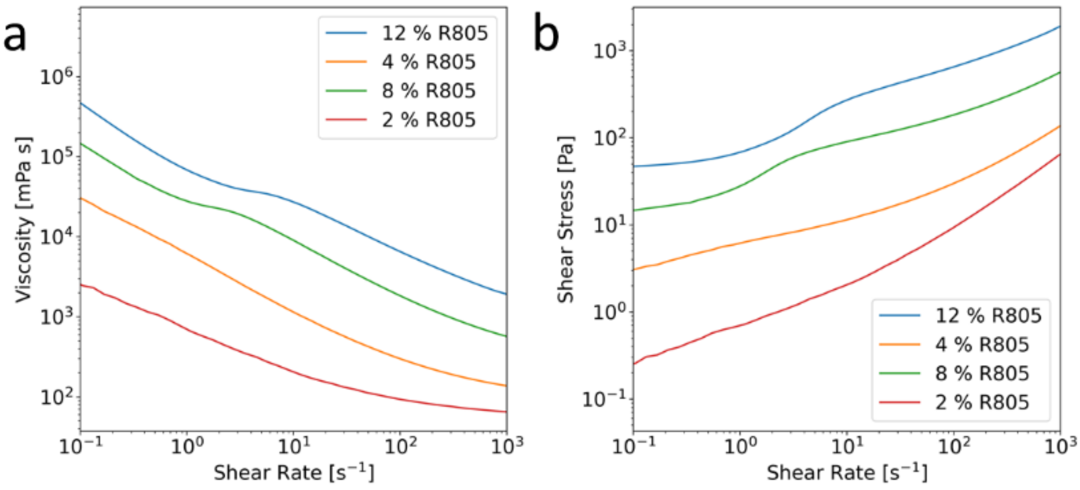

Supplementary Figure 2. Rheological characterization of the support bath for different R805 loadings. (a) Flow curves showing increased viscosity with increasing loading, all showing clear shear thinning behavior (b) Flow curves showing increasing shear stress for increasing R805 loading.

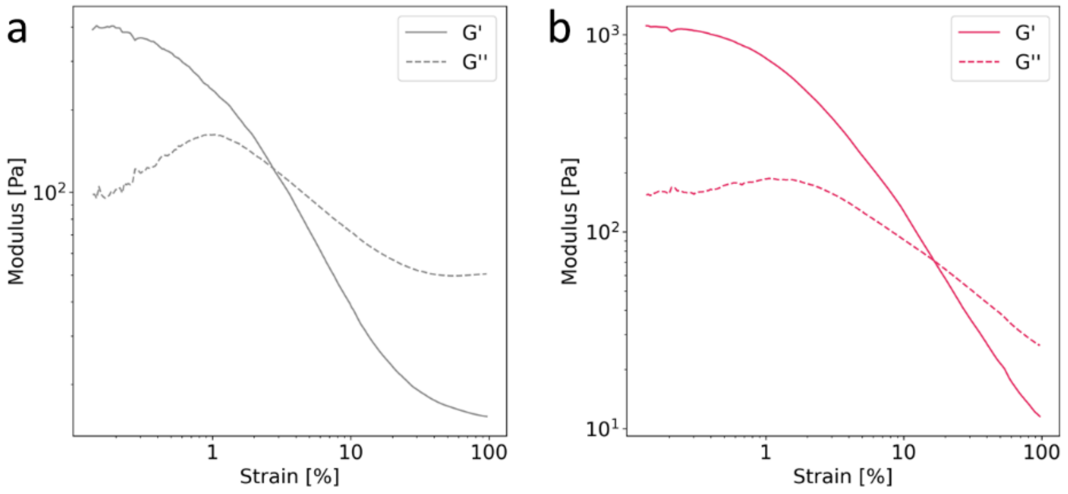

Supplementary Figure 3. (a) Amplitude sweep test for Mat 2. (b) Amplitude sweep test for Mat 1. Both show clear yielding behavior.

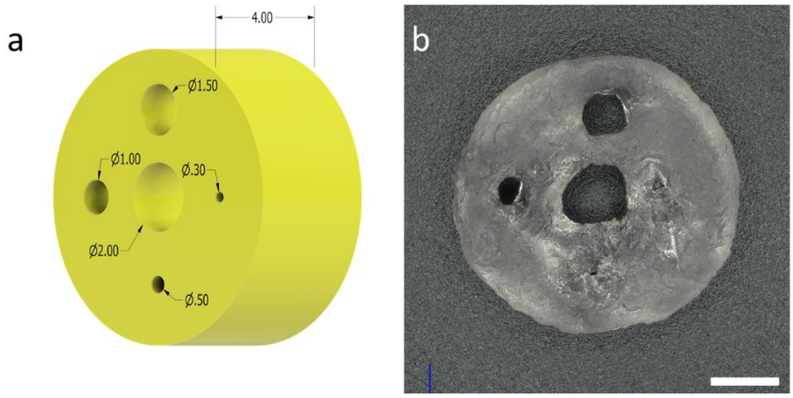

Supplementary Figure 4. Cylinder structure with 4 mm long straight channels of different diameters to test negative feature reproduction for volumetric printing. (a) 3D model of the test structure with diameters of vertical channels. Values are shown in mm. (b) Volumetrically printed sample showing clear overcuring starting from 0.5 mm diameter and fully clogged channels at 0.3 mm diameter. Scale bar (b): 2.2 mm

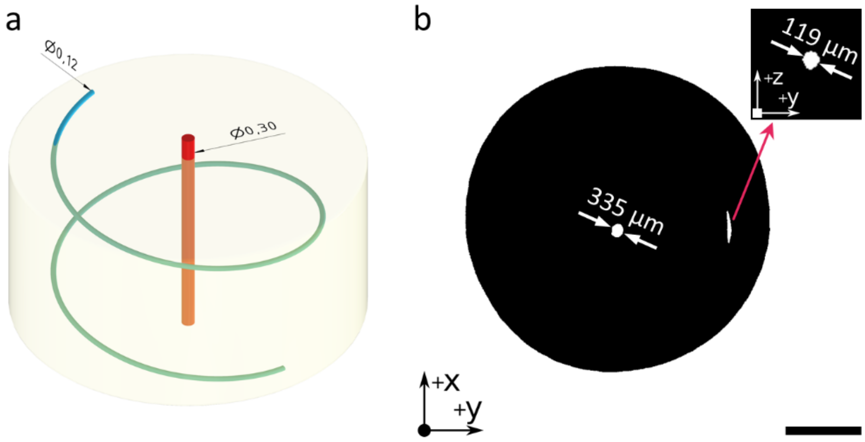

Supplementary Figure 5. Model and cross section of a cylindrical structure with integrated microchannles to showcase negative EmVP. (a) Model of the cylinder printed by TVAM and the helix and straight channels deposited by EMB3D, with modeled diameter of 120 µm and 300 µm respectively. (b) Cross sectional view along the XY plane of the printed part, showing a channel diameter of 335 µm for the straight channel. Inset shows the cross-sectional view along the YZ plane of the helical channel, with a diameter of 119 µm. The higher diameter for the vertical channel is due to the print path requiring the needle to move through the same spot twice. Channels embedded using a needle with a diameter of 150 µm. Scale bar (b): 2 mm

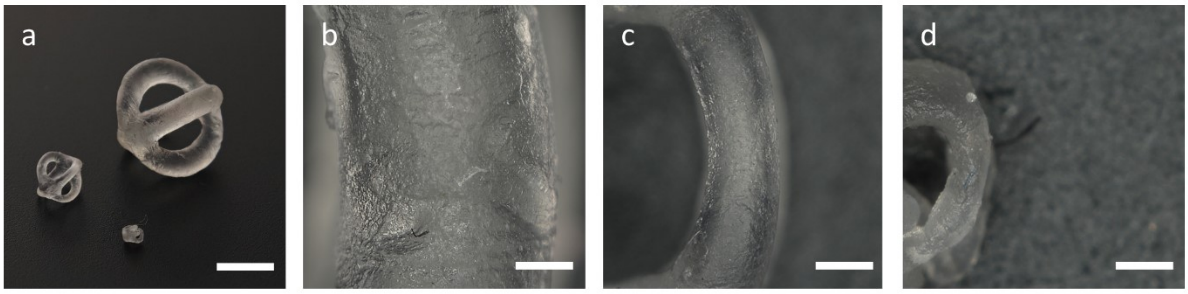

Supplementary Figure 6. Printing tests to assess resolution of the TVAM printer, showing decreasing sizes until first failures. (a) The printed structures, with external diameter of 10 mm, 5 mm and 2.5 mm respectively. (b) Microscope image of the beam for the 10 mm structure of (a), showing diameter of approx. 2 mm. (c) Microscope image of the beam for the 5 mm structure of (a), showing diameter of approx. 700 µm. (d) Microscope image of the beam for the 2.5 mm structure of (a), showing diameter of approx. 300 µm. At 2.5 mm external diameter obtaining an evenly resolved structure was not possible, as indicated by the uneven thickness of the spheres truss. Scale bars: (a) 5.5 mm, (b,c,d): 650 µm

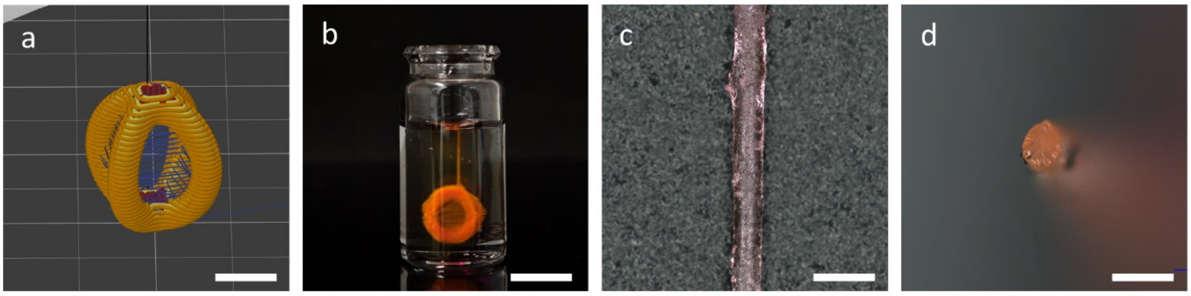

Supplementary Figure 7. EMB3D printing of a spherical cage structure and deposition of a single ink filament to demonstrate the resolution of the EMB3D printer. (a) Sliced model of the structure, showing travel lines in blue between the beams. (b) Deposited Mat 1 in a Mat 2 support bath according to the Gcode generated from the slicing in (a). Note visible layer lines and the presence of unintended connection between beams due to the inability of the EMB3D printer to completely stop material flow during travel movements. (c) Microscopy image of a printed filament, with height of 250 µm. (d) Microscopy image of a cross section of a printed filament, showing rounded shape with diameter of approx. 175 µm, corresponding to the minimum deposited filament diameter printable with our system for this ink/bath combination. Scale bars: a 4.2 mm, b 11 mm, c 500 µm, d 250 µm.

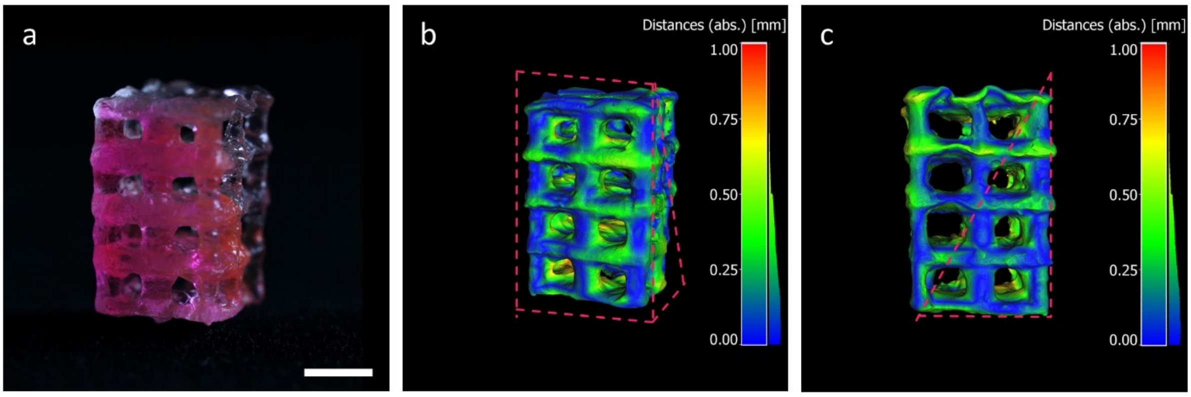

Supplementary Figure 8. Visualization of the printing accuracy for the multi-material structure of Figure 2g obtained by calculating the distance between the 3D model and the resulting part. (a) Printed positive EmVP structure. (b) CT scan of the structure of (a) colorized by the distance between the intended 3D model and a CT scan of the printed multi-material structure. Blue color indicates a match between the surface of the model and the print, while green yellow and red mark an increasingly high absolute distance of up to 1 mm (red). The data shows an average distance of 0.23±0.28 mm between model and print. The Mat 1 region is contained in the dashed lines (c) Frontal view of the CT scan of (b) with Mat 1 region contained in the dashed lines. Along the Z direction there is a gradual change from Mat 2 to Mat 1. The data shows that there is comparable deviation between model and print for both areas (Mat1 and Mat 2), which suggests a similar accuracy for the presented EmVP process when compared to classical single-material VAM. Scale bars: 4.3 mm

.

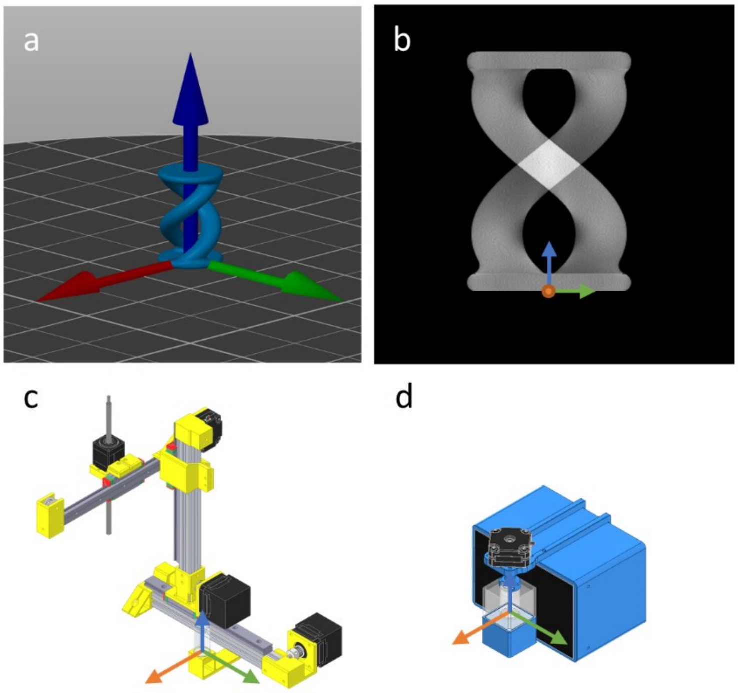

Supplementary Figure 9. Visualization of the origins of the reference frames used at different steps of the process. (a) Reference frame for the 3D model. (b) Reference frame for the projection pattern (c) Reference frame of the EMB3D printer (d) Reference frame of the TVAM printer. All reference frames are set to have the origin at the center of the 3D model in the XY plane and at the bottom of the model in the Z direction.

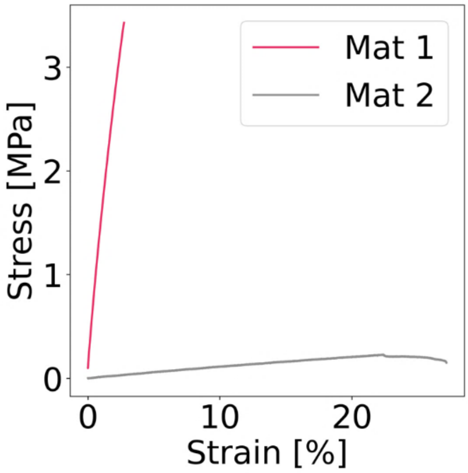

Supplementary Figure 10. Tensile test for cast sample of Mat 1 and Mat 2, showing very different mechanical properties. The elastic modulus is estimated to be 122 MPa for Mat 1 and 1.28 MPa for Mat 2.

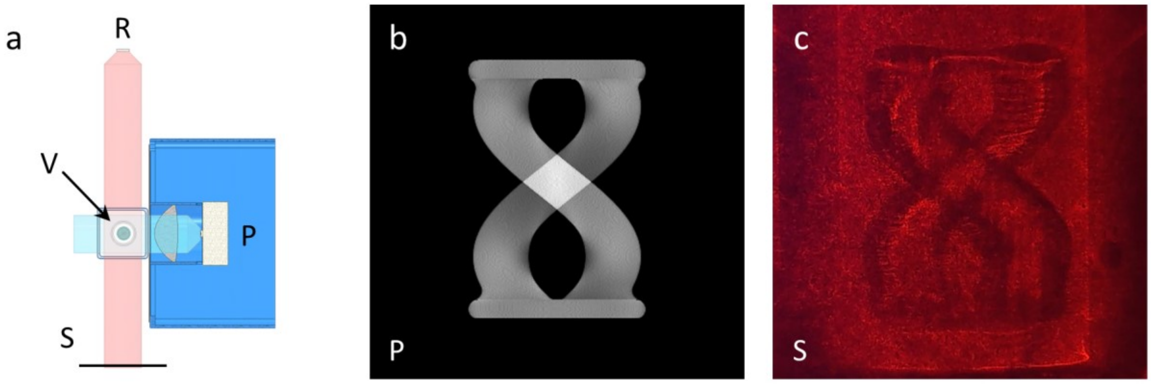

Supplementary Figure 11. Schematic representation of the TVAM print setup. (a) A red LED (R) casts a shadow of the vial (V) on a screen (S) orthogonally to the projection (P) propagation direction, with which the user determines print completion. (b) A projected pattern (c) Corresponding red-light shadowgram at print completion.

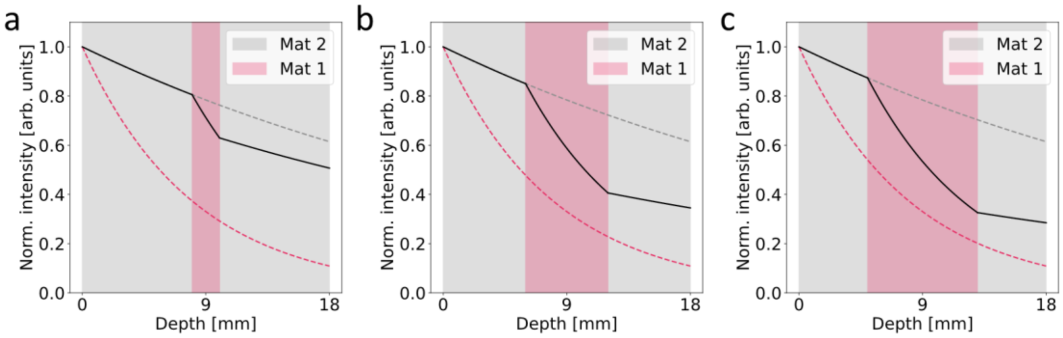

Supplementary Figure 12. Simulated intensity through the print volume for a vial with addition of different amounts of Mat 1 in Mat 2. (a) 2 mm long Mat 1 inclusion (b) 6 mm long Mat 1 inclusion (c) 8 mm long Mat 1 inclusion. Note how the intensity at the exit of the vial for the combined Mat1 and Mat2 volume is higher than the one for a full Mat 1 volume and for lower Mat 1 inclusion volumes, approximates the intensity of a fully Mat 2 filled vial. Dotted lines represent vials fully filled with Mat 1 or fully filled with Mat 2.
