# Supplementary material for: Additive manufacturing of multi-material and hollow structures by Embedded Extrusion-Volumetric Printing
Source: Nat Commun. 2025 Jul 22;16:6730. doi: 10.1038/s41467-025-62057-6 (PMC12284217; doi:10.1038/s41467-025-62057-6)
Supplement: Supplementary file 2 — Description of Additional Supplementary Files [file 41467_2025_62057_MOESM2_ESM.pdf]

Description of Additional Supplementary Files

File name: Supplementary Movie 1
Description: Compression of the skeleton sphere of Figure 2b, showing how the soft Mat 2 side compresses and bounces back while the stiff Mat 1 side is unaffected.

File name: Supplementary Movie 2
Description: Deflating and inflating of the bellow of Figure 2k, showing bending when a negative pressure is applied to the structure.

File name: Supplementary Code 1
Description: Python code for projection computation
